# Hemicellulases and auxiliary enzymes for improved conversion of lignocellulosic biomass to monosaccharides

**DOI:** 10.1186/1754-6834-4-5

**Published:** 2011-02-22

**Authors:** Dahai Gao, Nirmal Uppugundla, Shishir PS Chundawat, Xiurong Yu, Spencer Hermanson, Krishne Gowda, Phillip Brumm, David Mead, Venkatesh Balan, Bruce E Dale

**Affiliations:** 1 Biomass Conversion Research Lab (BCRL), Department of Chemical Engineering and Materials Science, Michigan State University, MBI Building, 3900 Collins Road, Lansing, Michigan 48910, USA; 2 Lucigen Corporation, 2120 West Greenview Drive, Middleton, Wisconsin 53562, USA; 3 Great Lakes Bioenergy Research Center (GLBRC), 164 Food Safety and Toxicology Building, Michigan State University, East Lansing, Michigan 48824, USA; 4 JiLin Rorgoo Renewable Energy Development Co Ltd, No.1 Sintang Rd, Jilin Econ and Tech Development Area, Jilin 132101, PR China

## Abstract

**Background:**

High enzyme loading is a major economic bottleneck for the commercial processing of pretreated lignocellulosic biomass to produce fermentable sugars. Optimizing the enzyme cocktail for specific types of pretreated biomass allows for a significant reduction in enzyme loading without sacrificing hydrolysis yield. This is especially important for alkaline pretreatments such as Ammonia fiber expansion (AFEX) pretreated corn stover. Hence, a diverse set of hemicellulases supplemented along with cellulases is necessary for high recovery of monosaccharides.

**Results:**

The core fungal cellulases in the optimal cocktail include cellobiohydrolase I [CBH I; glycoside hydrolase (GH) family 7A], cellobiohydrolase II (CBH II; GH family 6A), endoglucanase I (EG I; GH family 7B) and β-glucosidase (βG; GH family 3). Hemicellulases tested along with the core cellulases include xylanases (LX1, GH family 10; LX2, GH family 10; LX3, GH family 10; LX4, GH family 11; LX5, GH family 10; LX6, GH family 10), β-xylosidase (LβX; GH family 52), α-arabinofuranosidase (LArb, GH family 51) and α-glucuronidase (LαGl, GH family 67) that were cloned, expressed and/or purified from different bacterial sources. Different combinations of these enzymes were tested using a high-throughput microplate based 24 h hydrolysis assay. Both family 10 (LX3) and family 11 (LX4) xylanases were found to most efficiently hydrolyze AFEX pretreated corn stover in a synergistic manner. The optimal mass ratio of xylanases (LX3 and LX4) to cellulases (CBH I, CBH II and EG I) is 25:75. LβX (0.6 mg/g glucan) is crucial to obtaining monomeric xylose (54% xylose yield), while LArb (0.6 mg/g glucan) and LαGl (0.8 mg/g glucan) can both further increase xylose yield by an additional 20%. Compared with Accellerase 1000, a purified cocktail of cellulases supplemented with accessory hemicellulases will not only increase both glucose and xylose yields but will also decrease the total enzyme loading needed for equivalent yields.

**Conclusions:**

A diverse set of accessory hemicellulases was found necessary to enhance the synergistic action of cellulases hydrolysing AFEX pretreated corn stover. High glucose (around 80%) and xylose (around 70%) yields were achieved with a moderate enzyme loading (~20 mg protein/g glucan) using an in-house developed cocktail compared to commercial enzymes.

## Introduction

Lignocellulosic biomass, a world-wide abundant renewable feedstock [1-3], has the potential to be converted into fuels and chemicals [4-6]. Successful biological conversion of lignocellulosic biomass requires an efficient and economical pretreatment method, high glucose/xylose yields during enzymatic hydrolysis and fermentation of both hexose/pentose sugars to ethanol [5]. The prohibitively high cost of enzymes is a major hurdle to the implementation of economically feasible lignocellulosic biorefineries [7]. One of several strategies to decrease the cost of hydrolysis is by using tailor-made enzyme cocktails which contain all necessary activities with a defined protein composition [8-10].

Ammonia fiber expansion (AFEX), a leading pretreatment technology, is a 'dry to dry' process [7,11] with no wash or liquid stream being produced [7,12,13]. The biomass composition is almost identical after pretreatment [14]. Unlike other pretreatments, such as dilute acid, which extract a significant amount of hemicellulose [11], AFEX cleaves lignin-carbohydrate complexes without physically extracting hemicellulose or lignin into separate process streams [13,15,16]. Hence, efficient hydrolysis of AFEX treated biomass to achieve both high glucose and xylose yields requires supplementing the cellulases with hemicellulases and other accessory enzymes.

There have been numerous studies of enzymatic hydrolysis of pretreated biomass using commercial enzyme preparations. However, the limitations of using poorly characterized enzyme mixtures are obvious. First, it is difficult to evaluate the role of individual enzymes during hydrolysis without purifying the proteins from the mixture. Secondly, it is also difficult to vary the ratio of each component in order to obtain an optimal enzyme cocktail. Finally, using non-optimal commercial enzymes might lead to erroneous conclusions when comparing the effectiveness of pretreatments as the enzymes required for efficient hydrolysis are not identical for differentially pretreated biomass.

Our previous studies have allowed us to define the optimum ratio of six core fungal enzymes for AFEX treated corn stover. The cocktail consisted of cellobiohydrolases I and II (CBH I and CBH II), endoglucanase I (EG I), β-glucosidase (βG), endoxylanase (EX) and β-xylosidase (βX). More than 80% of theoretical glucose yield could be achieved using this optimized cocktail. However, irrespective of the amount of EX and βX loaded, xylose yield never exceed 56% [8], which suggests the inclusion of other hemicellulases and/or accessory enzymes is necessary in order to further increase xylose yields.

Glycosyl hydrolases from bacteria provide a plentiful source of enzymes which have the potential to be utilized in lignocellulose hydrolysis. Synergism between fungal cellulases and bacterial xylanases has been recently demonstrated in our previous studies [9]. In this work, we demonstrate that supplementing fungal cellulases and β-glucosidase (βG) with additional EXs and other hemicellulases can further increase the yields of glucose and xylose at reduced total enzyme loadings. Xylanases and two additional debranching hemicellulases from *Clostridium thermocellum, Geobacillus thermodenitrificans, G. stearothermophilus *and *Dictyoglomus turgidum *(cloned and expressed in *Escherichia coli*) along with fungal cellulases (CBH I, CBH II, EG I and βG purified from *Trichoderma reesei *and *Aspergillus niger *derived broths) were evaluated to test their hydrolytic efficacy on AFEX treated corn stover.

## Methods

### AFEX pretreatment

AFEX pretreatment of corn stover was carried out as described in our previous work [8]. A hybrid seed variety (33A14) based stover, provided by the National Renewable Energy Laboratory (USA), was harvested in 2002 from the Kramer farm in Wray (CO, USA) with 60% moisture (kg water/kg dry biomass) and transferred to a high pressure Parr reactor. Heated liquid ammonia (1 kg of ammonia/kg of dry weight biomass) was charged to the reactor vessel resulting in an immediate rise in temperature to 130°C. The reactor was maintained at 130°C for 15 min through an external heating mantle (within ± 10°C). At the end of 15 min, the pressure was reduced to an atmospheric level resulting in a precipitous drop in temperature of the reactor contents. The instantaneous pressure drop in the vessel caused the ammonia to vaporize, cooling the biomass to below 30°C. The pretreated material was left under the hood overnight to ensure the complete removal of any residual ammonia. AFEX pretreated corn stover was milled (Centrifugal mill ZM 200, Retsch, PA, USA) using a 0.1 mm sieve attachment as described elsewhere [17]. The composition of both the untreated and AFEX treated stover was approximately 34.4% glucan, 22.4% xylan and 11% lignin. Except for the addition of a small amount of ammonia and formation of certain decomposition products [15], gross biomass composition does not change due to AFEX.

### Discovery and cloning of endoxylanases (LX1 and LX2) and beta-xylosidase (LβX)

The details of discovery and cloning of LβX, LX1 and LX2 was based on previous work [9].

### Discovery and cloning of L-arabinofuranosidase (LArb)

Enrichment cultures were started from a grass compost and were grown in YTP-2 medium containing (per litre) 2.0 g yeast extract, 2.0 g tryptone, 2.0 g sodium pyruvate, 1.0 g KCl, 2.0 g KNO_3_, 2.0 g Na_2_HPO_4_.7H_2_O, 0.1 g MgSO_4_, 0.03 g CaCl_2_, and 2.0 mL clarified tomato juice. Enrichments were performed at 70°C in flasks agitated at 200 rpm. A number of aerobic cultures were purified by selection of individual colonies on plates containing the above medium and 16.0 g/L agar. For the preparation of genomic DNA, litre cultures were grown from a single colony in YTP-2 medium and collected by centrifugation. The cell was lysed using a combination of sodium dodecyl sulphate (SDS) and proteinase K and genomic DNA was isolated using a phenol/chloroform extraction [18]. The genomic DNA was precipitated, treated with RNase to remove residual contaminating RNA and fragmented by hydrodynamic shearing (HydroShear apparatus, Genomic Solutions, MI, USA) to generate fragments of either 3-5 kb or 10 kb. The fragments were purified on an agarose gel, end repaired and ligated into a high-stability, low copy vector (pSMART-LCKan, Lucigen, WI, USA). The recombinant plasmids were then used to transform *Escherichia coli *10G ELITE electrocompetent (Lucigen) cells and screened on plates containing 4-methylumbelliferyl-α-D-arabinopyranoside. DNA inserts of the positive clones were sequenced. The enzymes of interest were subcloned into pET28a and the resulting vectors used to transform BL21 (DE3) chemically competent cells. Amino acid sequences were deduced from the DNA sequences of the clones using ExPaSy translate tool (http://expasy.org/tools/dna.html) and confirmed by N-terminal sequencing/sodium SDS polyacrylamide gel electrophoresis (SDS-PAGE).

### Discovery and cloning of LX3, LX4, LX5, LX6 and LαGl

*D. turgidum *strain 6724T (Deutsche Sammlung von Mikroorganismen und Zellkulturen GmbH; German Collection of Microorganisms and Cell Cultures) bacterial cell concentrate was a kind gift of Dr Frank T Robb, Center of Marine Biotechnology, University of Maryland Biotechnology Institute, USA. *C. thermocellum *bacterial cell concentrate was a kind gift of Dr Paul Weimer, United States Department of Agriculture, Agricultural Research Service, United States Dairy Forage Research Center, WI, USA. The cell concentrate from each strain was lysed using a combination of SDS and proteinase K, and the genomic DNA was purified using a phenol/chloroform extraction methodology [18]. Genomic DNA was prepared as described in the previous section. *D. turgidum *and *C. thermocellum *genomic libraries were prepared as described in the previous section and screened on plates containing MUC. LX6 was obtained from screening of the *D. turgidum *library, and LX3 from screening of the *C. thermocellum *library. DNA inserts of the positive clones were sequenced, the enzymes of interest were subcloned into pET28a and the resulting vectors were used to transform BL21 (DE3) chemically competent cells [19]. Amino acid sequences were deduced from the DNA sequences of the clones using ExPaSy translate tool (http://expasy.org/tools/dna.html) and confirmed by N-terminal sequencing/SDS PAGE. LX4 and LX5 were cloned by polymerase chain reaction (PCR) amplification of the desired genes from *C. thermocellum *genomic DNA, while LαGl was cloned by PCR amplification of the desired gene from *D. turgidum *genomic DNA.

### Enzyme expression and purification

Plasmids containing the specific protein genes (LX3, LX4, LX5, LX6, LArb and LαGl) were transformed into *E. coli *BL21 (DE3) cells for protein expression. A starter culture, started from frozen stocks, was grown overnight by incubating at 37°C in kanamycin (30 μg/mL) + 0.4% glycerol. Flasks of LB soy + kanamycin + 0.4% glycerol were started with the overnight culture and grown to an OD600 of 0.7-0.8, induced with 1 mM IPTG (isopropylthio-β-D-galactoside) and then incubated overnight (16-18 h) at 37°C. Cells were harvested by centrifugation (4000 rpm, 30 min) and the supernatant removed. Cell pellets were resuspended in lysis buffer (50 mM Tris-HCl, pH 8.0, 300 mM NaCl for His6 samples or 50 mM Tris-HCl, pH 8.0, for non-tagged samples) and sonicated using a sonics vibra cell large tip. The mixture was centrifuged at 12,000 rpm for 30 min to remove the cell debris. The supernatant was heated to 60°C for 20 min and centrifuged again at 12,000 rpm to further remove precipitated debris. For proteins containing a His6 tag, the supernatant was filtered through 0.5 μm filter and applied on 40 mL bed volume HIS-select nickel affinity gel (Sigma, MO, USA) equilibrated in lysis buffer. The column was washed with a minimum of 10 column volumes of lysis buffer. The bound protein was eluted using six column volumes of elution buffer (25 mM Tris-HCl, pH 8.0, 300 mM NaCl, 300 mM Imidazole). For proteins not containing a His6 tag, the supernatant was filtered through 0.5 μm filter and applied on 20 mL bed volume Q Sepharose Fast Flow (GE Healthcare, NJ, USA) equilibrated in 50 mM Tris-HCl, pH 8.0 and then washed with 10 column volumes (200 mL) of Q-Sepharose wash buffer (25 mM Tris-HCl, pH 8.0, 25 mM NaCl. The enzyme was eluted using a 200 mL gradient of 50 to 500 mM NaCl in 25 mM Tris-HCl, pH 8.0. Enzyme purity was verified on Pierce 4-20% SDS-PAGE (Thermo Fisher Scientific, IL, USA). The enzyme samples were concentrated using Amicon Ultra-15 membrane, dialyzed against storage buffer (50 mM Tris-HCl, pH 7.5, 100 mM NaCl and 20% glycerol) and quantified using the Pierce Bradford assay kit (Thermo Fisher Scientific) with bovine serum albumin as the standard. The amino acid sequences and glycosyl hydrolase families for all six bacterial enzymes are shown in Table 1.

**Table 1:** Amino acid sequences and glycosyl hydrolase families for all six bacterial enzymes.

Notation	Amino acid sequence	GH family	**Uniprot No**.	Source, name and predicted molecular weight
**LX3**	MSGNALRDYAEARGIKIGTCVNYPFYNNSDPTYNSILQREFSMVVCENEMKFDALQPRQNVFDFSKGDQLLAFAERNGMQMRGHTLIWHNQNPSWLTNGNWNRDSLLAVMKNHITTVMTHYKGKIVEWDVANECMDDSGNGLRSSIWRNVIGQDYLDYAFRYAREADPDALLFYNDYNIEDLGPKSNAVFNMIKSMKERGVPIDGVGFQCHFINGMSPEYLASIDQNIKRYAEIGVIVSFTEIDIRIPQSENPATAFQVQANNYKELMKICLANPNCNTFVMWGFTDKYTWIPGTFPGYGNPLIYDSNYNPKPAYNAIKEALMGYHHHHHH	**10**	P10478	Truncated *Clostridium thermocellum* (XynZ, 38.0 kDa) (corresponds to last 324 A.A. in protein of the mature enzyme)
**LX4**	MKMGKMYEVALVVEGYQSSGKADVTSMTITVGNAPSTSSPPGPTPEPTPRSAFSKIEAEEYNSLKSSTIQTIGTSDGGSGIGYIESGDYLVFNKINFGNGANSFKARVASGADTPTNIQLRLGSPTGTLIGTLTVASTGGWNNYEEKSCSITNTTGQHDLYLVFSGPVNIDYFIFDSNGVNPTPTSQPQQGQVLGDLNGDKQVNSTDYTALKRHLLNITRLSGTALANADLNGDGKVDSTDLMILHRYLLGIISSFPRSNPQPSSNPQPSSNPQPTINPNAKLVALTFDDGPDNVLTARVLDKLDKYNVKATFMVVGQRVNDSTAAIIRRMVNSGHEIGNHSWSYSGMANMSPDQIRKSIADTNAVIQKYAGTTPKFFRPPNLETSPTLFNNVDLVFVGGLTANDWIPSTTAEQRAAAVINGVRDGTIILLHDVQPEPHPTPEALDIIIPTLKSRGYEFVTLTELFTLKGVPIDPSVKRMYNSVP	**11**	O87119	Truncated *C. thermocellum* (XynA, 52.1 kDa) (corresponds to residues 199-683 of the mature enzyme)
**LX5**	KNKRVLAKITALVVLLGVFFVLPSNISQLYADYEVVHDTFEVNFDGWCNLGVDTYLTAVENEGNNGTRGMMVINRSSASDGAYSEKGFYLDGGVEYKYSVFVKHNGTGTETFKLSVSYLDSETEEENKEVIATKDVVAGEWTEISAKYKAPKTAVNITLSITTDSTVDFIFDDVTITRKGMAEANTVYAANAVLKDMYANYFRVGSVLNSGTVNNSSIKALILREFNSITCENEMKPDATLVQSGSTNTNIRVSLNRAASILNFCAQNNIAVRGHTLVWHSQTPQWFFKDNFQDNGNWVSQSVMDQRLESYIKNMFAEIQRQYPSLNLYAYDVVNEAVSDDANRTRYYGGAREPGYGNGRSPWVQIYGDNKFIEKAFTYARKYAPANCKL YYNDYNEYWDHKRDCIASICANLYNKGLLDGVGMQSHINADMNGFSGIQNYKAALQKYINIGCDVQITELDISTENGKFSLQQQADKYKAVFQAAVDINRTSSKGKVTAVCVWGPNDANTWLGSQNAPLLFNANNQPKPAYNAVASIIPQSEWGDGNNPAGGGGGGKPEEPDANGYYYHDTFEGSVGQWTARGPAEVLLSGRTAYKGSESLLVRNRTAAWNGAQRALNPRTFVPGNTYCFSVVASFIEGASSTTFCMKLQYVDGSGTQRYDTIDMKTVGPNQWVHLYNPQYRIPSDATDMYVYVETADDTINFYIDEAIGAVAGTVIEGPAPQPTQPPVLLGDVNG	**10**	B4BME8	Truncated *C. thermocellum* (XynY, 81.4 kDa) (corresponds to residues 1-736 of mature enzyme)
**LX6**	MEIPSLKEVYKDYFPIGAAVSHLNIYTYEDLLKKHFNSLTPENQMKWEVIHPKPYVYDFGPADEIVDFAMKNGMKVRGHTLVWHNQTPGWVYAGTKDEILARLKEHIYEVVGHYKGKVYAWDVVNEALSDNPNEFLRKAPWYDICGEEVIEKAFIWANEADPNAKLFYNDYNLEDPIKREKAYQLVKRLKEKGIPIHGVGIQGHWTLAWPTPKMLEDSIKRFSELGVEVQITEFDISIYYDRNENNNFKVPPDDRIEKQAQLYKQAFEILRKYRGVVTGVTFWGVADDYTWLYFWPVRGREDYPLLFDKNHNPKKAFWEIVKFHHHHHH	**10**	B8E346	*Dictyoglomus turgidum* (Dtur_1647, 38.1 kDa)
**LαGl**	MAEVKPYNMCWLEYTDLSKYKNKYIKVFENVVVLGGNELNLPLKELKNFLTFSLNIKPKIFKNTLVKGRNYVLIGRLIEIKKIFKESERFEKLLNDEGFIIKRIDIDGNKVLIITAKSYNGIVYGIFNLIERLKRGEDIENIDIVSNPSLRFRMLNHWDNLDGSIERGYAGKSIFFRENKILINERTKDYARLLSSIGVNGVVINNVNVKKKEVELITPSYLKKIGELSKIFSAYGIKIY LSINFASPIYLGGLNTADPLDKRVAVWWKAKVDEIYEYVPDFGGFLVKADSEFNPGPHMYGRTHADGANMLGEALESYGGFVIWRAFVYNCLQDWRDTNTDRAKAAYENFKPLDGKFSENVIVQIKYGPMDFQVREPVNPLFGGLEHTNQILELQITQEYTGQQIHLCYLGTLWKEVLDFDTYAKGEGSKVKEILKGNVFDLKNNGMAGVSNVGDDINWTGHDLAQANLYTFGALSWNPDERIEEVVKRWIELTFGDNEKVIKNISYMLLSSHKAYEKYTTPLGLGWMVNPGHHYGPNPEGYEYSKWGTYHRANYEAIGVDRSSRGTGYTLQYHSPWREIYDNIETCPEELLLFFHRVPYNYKLKSGKTLIQTYYDLHFEGVEEAEEIRKKWIELKGEIEDKIYERVLNRLDIQIEHAKEWRDVINTYFFRRTGIPDEKGRKIYPHHHHHH	**67**	B8E3B2	*D. turgidum* (Dtur_1714, 79.4 kDa)
**LArb**	MSVKRAQMIIEKDFKIAKIDKRIYGSFIEHLGRAVYGGIYEPGHPQADENGFRQDVIEMVKELQVPIIRYPGGNFVSGYNWEDGVGPKEKRPRRLDLAWKSVETNEIGLNEFVDWAKMVGAEVNMAVNLGTRGIDAARNLVEYCNHPSGSYYSDLRISHGYKEPHKIKTWCLGNEMDGPWQIGHKTAVEYGRIACEAAKVMKWVDPTIELVACGSSGRNMPTFAEWEAIVLDHTYEHVDYISLHQYFGNRDNDTANYLAMSLEMDDFIRSVVAIADYVKAKKRSKKTIHLSFDEWNVWYHSNEADKQIEPWTVAPPLLEDIYNFEDALLVGCMLITLMKHADRVKIACLAQLVNVIAPIM TEKNGPAWKQTIYYPFMHASVYGRGVALHPVISSPKYDSKDFTDVPYLESIAVYNEEKEEVTIFAVNRDMEDSLLLECDVRNFEDYRVIEHIVLEHENVKQTNSAQSSPVVPHRNGNAQM	**51**	B4BMB8	*Geobacillus sp. *G11MC16 (57.1 kDa)

Purification of fungal enzymes was performed using an FPLC system (GE Healthcare, Buckinghamshire, UK) at room temperature, while the fraction collector was refrigerated. Cellulases (CBH I, CBH II and EG I) and βG were purified from Accellerase (Genencor, NY, USA) and Novo 188 (Novozyme, CA, USA), respectively. Details of the purification methodology are described in our previous work [8].

### Enzymatic hydrolysis of pretreated biomass

The hydrolysis experiments were performed in 2.2 mL deep well microplates (Greiner, NC, USA) at 0.2% (w/w) total glucan loading along with 50 mM citrate buffer (pH 4.5) in a total volume of 500 μL per well, based on the microplate hydrolysis protocol described previously [17]. The enzymes mixtures were prepared separately and added simultaneously using a single or eight-channel automated pipetting workstation (epMotion, Hamburg, Germany) and 96-channel automated pipetting workstation (JANUS, Perkin Elmer, MA, USA). The microplates were incubated at 50°C with shaking at 250 rpm for 24 h. The concentrations of glucose and xylose in the hydrolyzates were measured using suitable enzyme based assays described below. All experiments were carried out in triplicates.

### Glucose and xylose assays

Glucose and xylose concentrations were measured using enzymatic kits purchased from R-Biopharm (MI, USA) and Megazyme (Bray, Ireland), respectively. D-Glucose is first phosphorylated to D-glucose-6-phosphate using ATP and hexokinase. The reaction of D-glucose-6-phosphate with NADP^+ ^is catalyzed by glucose-6-phosphate dehydrogenase to form D-gluconate-6-phosphate and NADPH. The reactions are stoichiometric to the amount of D-glucose and a corresponding increase in NADPH is measured at 340 nm to estimate the glucose concentration. The xylose assay is based on analogous two-step reactions. α-D-xylose is converted to isomeric β-D-xylose by xylose mutarotase. β-D-xylose is then reacted with NAD^+^, in presence of β-xylose dehydrogenase, to form D-xylonic acid and NADH. The corresponding increase of NADH is measured at 340 nm to determine the xylose concentration. The entire protocol (including liquid transfers and mixing), was performed using an automated 96-channel pipetting workstation (Janus, PerkinElmer, CA, USA). Based on the information provided by the manufacturer, β-xylose dehydrogenase has no cross-reactivity on L-arabinose.

## Results and discussion

### Supplementing bacterial hemicellulases along with fungal cellulases

**Figure 1:**
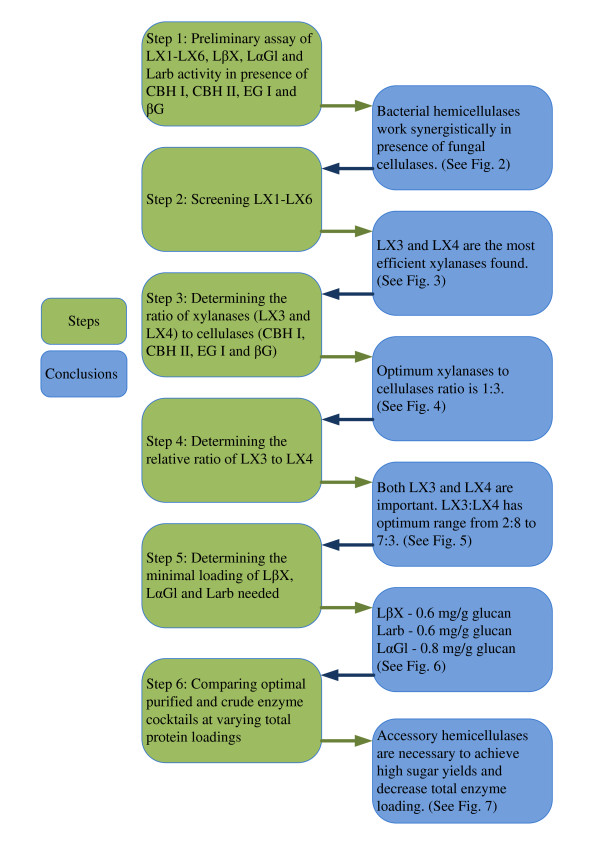
**The overall conclusions drawn from each step of the process of hemicellulases optimization in the presence of cellulases during hydrolysis of ammonia fiber expansion (AFEX) treated corn stover**.

Previous work has shown that *Dictyoglomus *and *Clostridium *derived hemicellulases retain significant activity at pH 4.5-5, whereas *Trichoderma *derived cellulases lose considerable activity at pH greater than five [9]. Hence, all assays were conducted at pH 4.5-5 to maximize performance of both sets of enzymes. Overall strategy used to obtain an optimized cocktail and major findings from this study are highlighted in Figure 1. From step 2 onwards, each experimental step was designed and tested based on conclusions from preceding steps. For step 1, to test performance of the nine different hemicellases on AFEX treated corn stover, all six xylanases (4 mg/g glucan for each xylanase) and other three accessory enzymes (2 mg/g glucan for each of LβX, LαGl and LArb, respectively) were grouped and doped together along with the core cellulases mixture. This avoided the risk of missing any synergism among the endoxylanases (LXs) and also between LXs and accessory enzymes (LβX, LαGl and LArb). Core fungal enzymes consisted of CBH I, CBH II and EG I (4 mg/g glucan loading of each cellulase) plus 2 mg/g glucan βG loading. Previous results have shown the current βG loading is sufficient to prevent cellobiose build-up at the current glucan loading [8]. The protein mass ratio of CBH I, CBH II and EG I was kept at 1:1:1 in this study since previous results have demonstrated that this ratio results in optimum activity [9]. The minimum loading of LβX, LαGl and LArb required was determined after screening for optimal endoxylanase combination.

**Figure 2:**
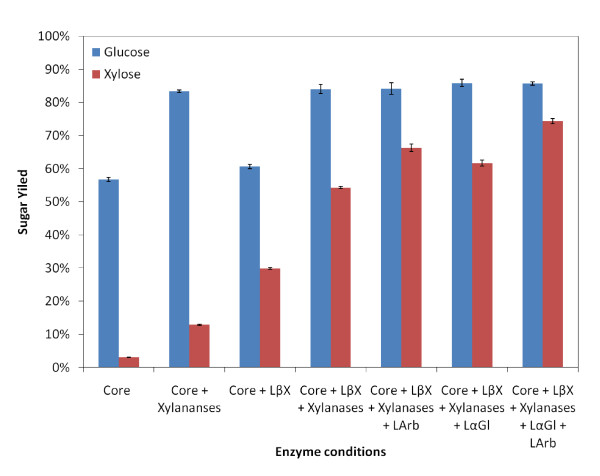
**The glucose (blue bar) and xylose (red bar) yields after 24 h hydrolysis of ammonia fiber expansion (AFEX)-treated corn stover for different enzyme cocktails**. Core enzymes only contain CBH I, CBH II, EG I (4 mg/g glucan each) and βG (2 mg/g glucan). Endoxylanases include LX1, LX2, LX3, LX4, LX5 and LX6 (4 mg/g glucan each). Accessory hemicellulases LArb, LαGl and LβX were loaded at 2 mg/g glucan each.

From Figure 2, core cellulases alone account for 56% glucose yield and minimal (3%) xylose yield within 24 h hydrolysis. Supplementing xylanases along with core cellulases increased glucose yield to as high as 83%. However, only 13% xylose yield was achieved. One possible reason for the improvement in glucan conversion is that xylanase supplementation helps remove xylan [20] sheathing cellulose fibrils and, hence, increases substrate accessibility [21]. LβX supplementation allows cleavage of xylo-oligomers to monomeric xylose and hence mixtures containing LβX have higher monomeric xylose yield. The α arabinofuranosidase removes arabinose side-chains, while α-glucuronidase cleaves the α 1,2-glycosidic bond of the 4-O-methyl-D-glucuronic acid side chain from the xylan backbone [22,23]. Adding LArb and LαGl along with cellulases and xylanases, further increased xylose yields by 12% and 7%, respectively. Adding both these enzymes, along with core cellulases and xylanases, allowed the xylose yield to reach as high as 74%. Hence, the removal of side chains which impede endoxylanase activity could enhance xylose yields significantly [24-26]. However, the total enzyme loading employed to obtain this conversion was 44 mg/g glucan, which is not viable for industrial processing. Therefore, systematically screening the critical endoxylanases and determining the minimum amounts of accessory enzymes is necessary to decrease the total enzymes loading without sacrificing hydrolysis performance.

### Screening endoxylanases

In Figure 2, all 6 xylanases were loaded together to evaluate their performance along with LβX, LαGl and LArb and also to prevent the risk of missing any synergism among the xylanases. However, not all xylanases may be necessary to bring about efficient xylan hydrolysis. In order to determine the most important xylanases or their combinations, a two level (0 and 4 mg/g glucan for low and high level of enzymes loading), six factor (LX 1 to LX 6) full factorial experiment was carried out by fixing the remaining enzyme loading (CBH I, CBH II and EG I at 4 mg/g glucan each; βG, LβX, LαGl and LArb at 2 mg/g glucan each).

**Figure 3:**
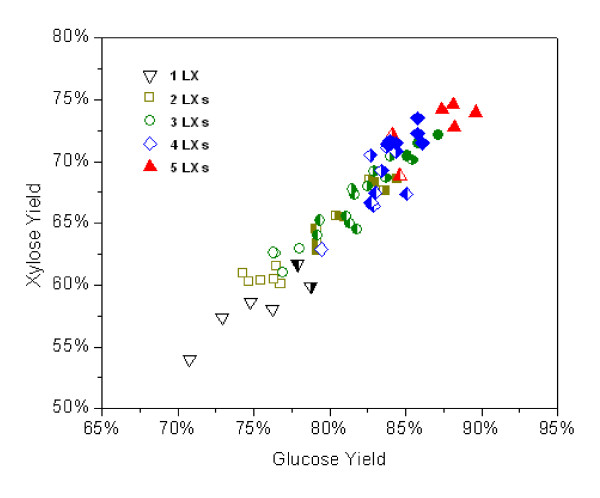
**The glucose and xylose yields after 24 h hydrolysis of ammonia fiber expansion (AFEX)-treated corn stover for different xylanase loadings**. LX1 to LX6 were loaded as different combinations (from single enzyme loading to five enzymes added simultaneously). Open symbol denotes that no LX3 or LX4 were included in the mixture. The left half-filled symbol denotes that LX3 was included in the mixture. The right half-filled symbol denotes LX4 was included. The closed symbol denotes both LX3 and LX4 were present in the mixture.

Results of the 24 h hydrolysis results for AFEX corn stover are shown in Figure 3 which depicts the glucose versus xylose yields. The xylose yield shows a strong linear relationship with the glucose yield. This reconfirmed previous findings that high glucose yields can be achieved at higher xylose yields for AFEX treated corn stover [9]. In this experiment, xylanase loading increased depending on the number of xylanases involved in the mixture. Experiments performed by adding individual xylanases showed that LX3 gave the highest conversion followed by LX4. For binary LX mixtures, the best mixture contained both LX3 and LX 4. Mixtures containing neither of these two enzymes resulted in 10% or more drop in both glucose and xylose yield. Combinations containing either LX3 or LX4 exhibited moderate conversions. For ternary mixtures, and other higher multiple LX loadings, LX3 and LX4 gave a similar trend which suggests that both LX3 and LX4 are superior compared to other xylanases.

### Optimum cellulase to xylanase ratio

Based on previous results, both LX3 and LX4 were chosen as supplementary xylanases for further studies. As xylose and glucose yields are highly correlated, the ratio between cellulases and xylanases loaded was further optimized.

**Figure 4:**
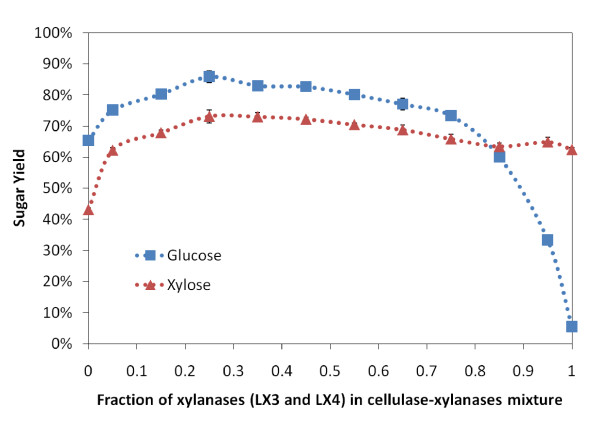
**The glucose (blue) and xylose (red) yields after 24 h hydrolysis of ammonia fiber expansion (AFEX)-treated corn stover for different ratios of endoxylanases LX3 and LX4 in total enzymes (CBH I, CBH II, EG I, LX3 and LX4) loading of 20 mg/g glucan**. All enzymes were loaded at equi-mass ratio, except βG, LβX, LαGl and LArb that were additionally supplemented at 2 mg/g glucan each.

In Figure 4, varying ratios of xylanases (LX3 and LX 4 at equi-mass loading) were supplemented along with fungal core cellulases (CBH I, CBH II and EG I at equi-mass loading) for a fixed total protein loading of 20 mg/g glucan. A fixed amount (2 mg/g glucan) of other enzymes (βG, LβX, LaGl and LArb) were also loaded. Both the glucose and the xylose yields are shown in Figure 4. Without any xylanases, the glucose and xylose yields dropped to 63% and 43%, respectively. As EG I has some xylanase activity [8,27], even in the absence of any other xylanases, a significant xylose yield was obtained by the endocellulase in the presence of LβX. Supplementing the mixture with 5% xylanase increased both the glucose and xylose yields significantly. At 25% xylanase supplementation, the highest observed glucose and xylose yields were seen. When no cellulases were loaded, the glucose yield drops to less than 10%. Interestingly, xylanase loadings, ranging from 15% to 75% of total enzyme added, gave comparable glucose (>70%) and xylose yields (>60%).

### Synergism between endoxylanases (LX3 and LX4)

**Figure 5:**
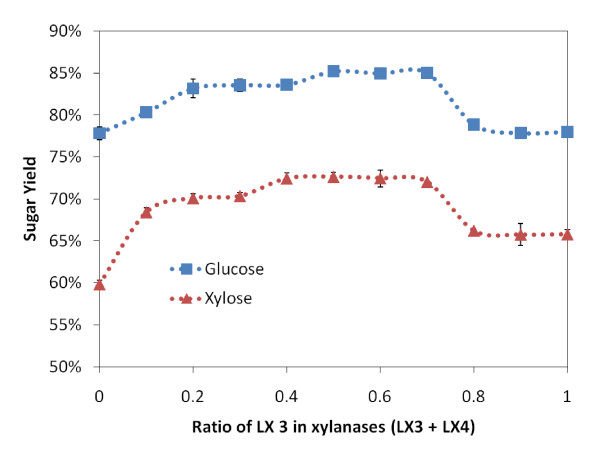
**The glucose (blue) and xylose (red) yields after 24 h hydrolysis of ammonia fiber expansion (AFEX)-treated corn stover for different ratios of LX3 and LX4**. We added 5 mg/glucan of each CBH I, CBH II, EG I and (LX3 +LX4) to the hydrolysis. Other enzymes (βG, LβX, LαGl and LArb) were each added at 2 mg/g glucan.

The optimal endoxylanase loading is one-third of the amount of cellulases added for hydrolysis. It is interesting to compare the synergistic role of each xylanase in the mixture. Based on previous results, cellulases (CBH I, CBH II and EG I) were loaded at 5 mg/g glucan each, xylanases (LX3 and LX4) were loaded at 5 mg/g glucan total and accessory enzymes (βG, LβX, LαGl and LArb) were loaded at 2 mg/g glucan each. Several reports highlight the synergism between exo-glucanases and also between different families of hemicellulases [28-30]. It is interesting to determine if there is similar synergism between the endoxylanases. Figure 5 shows that supplementing only LX4 or LX3 along with core cellulases and accessory enzymes results in xylose yields of 60% and 65%, respectively. However, synergistic combinations of LX3 and LX4 resulted in a greater than 70% xylose yield. In addition, the glucose yield also benefit from including both LX3 and LX4. Table 1 shows that LX3 and LX4 belong to GH family 10 and 11, respectively. These results suggest that both family 10 and 11 glycosyl hydrolases are necessary to achieve complete xylan conversion for AFEX treated corn stover. It is normally believed that family 10 xylanases are more versatile and efficient than family 11 xylanases [24,28]. Family 10 xylanases cleave β-1,4 linkages from at least one unsubstituted xylopyranosyl residue adjacent to substituted xylopyranolsyl residues towards the reducing end. However, family 11 xylanases only cleave linkages adjacent to at least two unsubstituted xylopyranosyl residue [24]. Our results are also consistent with Banerjee *et al*., who found that a combination of GH family 10 and 11 fungal xylanases help increase both glucose and xylose yields [25]. Ratios of LX3/(LX3+LX4) between 0.4-0.7 yielded comparable total monosaccharides yields. These results reconfirm previous observations that both LX3 and LX4 are required in order to achieve higher monosaccharides yields.

### Minimizing loading of accessory enzymes (LβX, LαGl and LArb)

**Figure 6:**
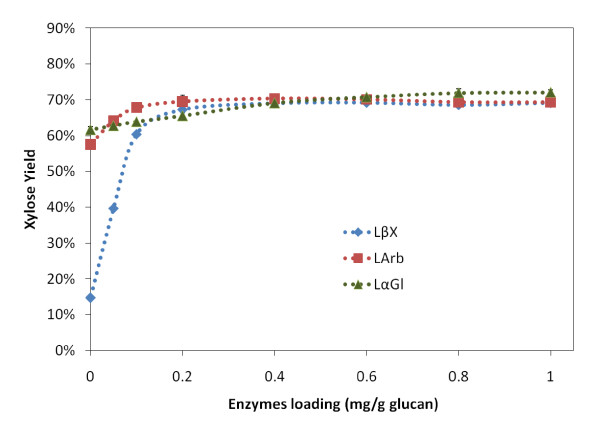
**The xylose yield after 24 h hydrolysis of ammonia fiber expansion (AFEX)-treated corn stover in the presence of accessory enzymes**. LβX, LArb and LαGl were loaded individually at different protein loadings, in the presence of an excess of the other two enzymes (1 mg/g glucan each). Major cellulases (CBH I, CBH II and EG I), hemicellulases (LX3 and LX4) were loaded at 5 and 2.5 mg/g glucan each, respectively. Additional βG (2 mg/g glucan) was added to prevent cellobiose build-up.

In all of the above studies, LβX, LαGl and LArb loading was in excess. In order to determine the minimum loading of each enzyme, different amounts of each were loaded while the other two were kept in excess. Glucose yields for all experiments were comparable (data not shown) which suggests that these enzymes do not influence glucan hydrolysis. However, there was a significant change in the xylose yields as shown in Figure 6. The minimum loading for LβX, LαGl and LArb was 0.6, 0.8 and 0.6 mg/g glucan, respectively. Note: LβX plays a very important role on xylose yield. Without LβX, the xylose yield is very low, even when LαGl and LArb are loaded.

### Comparison of monosaccharides yield for optimized and crude commercial enzyme preparations

**Figure 7:**
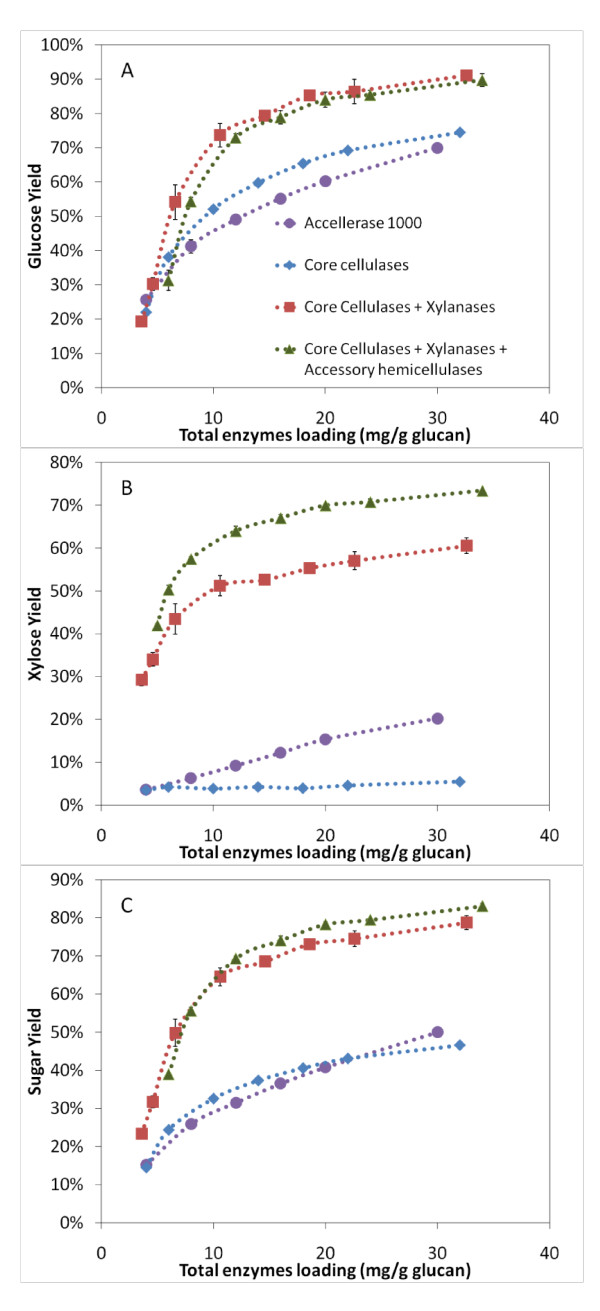
**The glucose (A), xylose (B) and total monosaccharide (C) yields after 24 h hydrolysis of ammonia fiber expansion (AFEX)-treated corn stover for various enzyme cocktails**. Accellerase 1000 is a commercial preparation. Core cellulases contain CBH I, CBH II and EG I at equi-mass loading along with 2 mg/g glucan βG. The xylanases (LX3 and LX4 at equi-mass ratio) to cellulases (CBH I, CBH II and EG I) ratio is fixed at 1:3. LβX, LαGl and LArb were loaded at 0.6, 0.8 and 0.6 mg/g glucan, respectively).

The previous experiments helped define the optimal cellulase and hemicellulase loadings necessary to maximize monomeric glucose and xylose yields from AFEX treated corn stover. It was interesting to compare the hydrolytic activity of optimized cellulase-hemicellulase cocktail with purified core cellulases and crude commercially available enzymes on pretreated biomass with respect to the maximum monosaccharide yield achieved. In Figure 7, four different enzyme mixtures were compared at varying total protein loadings for glucose (Figure 7A), xylose (Figure 7B) and total monosaccharide (Figure 7C) yield. The comparisons were made against: a commercial enzyme preparation (Accellerase 1000, Genencor, NY, USA); a core cellulase cocktail (CBH I, CBH II and EG I at equi-mass loading, along with 2 mg/g glucan βG); a core cellulase plus xylanase cocktail (3:1 mass ratio between cellulases to xylanases, 2 mg/g glucan βG and 0.6 mg/g glucan LβX); and an optimized cellulase-hemicellulase cocktail (core cellulase-xylanase cocktail, along with LαGl and LArb loaded at 0.8 and 0.6 mg/g glucan, respectively).

From Figure 7, at a fixed total protein loading (30 mg/g of glucan), it is clear that the commercial enzyme (Accellerase 1000) has limited hydrolytic activity on both glucan (70%) and xylan (20%) fractions for AFEX treated corn stover due to the lack of suitable hemicellulase activity. The core cellulase mixture (CBH I, CBH II and EG I) had slightly better glucan conversions but lower xylan yields (<5%) compared to the commercial preparations. Interestingly, the inclusion of xylanases and xylosidases enhanced both glucose and xylose yields significantly. When small amounts of accessory hemicellulases (LαGl and LArb) were included in the core cellulase-xylanase mixtures, glucose yields were unchanged while xylose yields increased to >70%. For the total monosaccharide yield (Figure 7C), Accellerase 1000 at 30 mg/g glucan loading has a similar yield (50%) compared to the optimized cocktail at around 7 mg/g glucan.

In order to achieve monosaccharides yields greater than 80%, which is necessary for an economical industrial process, the optimized cocktail concentration needed to be around 22 mg/g glucan while the non-optimal cellulase-xylanase mixtures required more than 33 mg/g glucan. These results demonstrate how trace amounts of important accessory hemicellulases can further enhance the overall polysaccharide hydrolysis yields for AFEX pretreated corn stover. About 1.2 mg/g glucan total loading of LArb and LαGl can decrease the overall enzyme loading by over 33%. Furthermore, if the cocktail does not contain specific activities, such as β-xylosidase, α-arabinofuranosidase and α-glucuronidase, no matter how much of the other enzymes are loaded, it is impossible to achieve high xylose hydrolysis yields.

## Conclusion

In this work, an optimized cocktail of xylanases and accessory enzymes was identified for AFEX treated corn stover. This cocktail included both fungal cellulases (CBH I, CBH II, EG I and βG) and bacterial hemicellulases (LX3, LX4, LArb, LαGl and LβX). The optimized cocktail can hydrolyze AFEX treated corn stover, resulting in glucose and xylose hydrolysis yields greater than 80% and 70%, respectively, at a reasonable protein loading (~20 mg/g glucan). Adding endoxylanases increases both xylose and glucose yields significantly (along with a suitable β-xylosidase). Supplementation of accessory α-arabinofuranosidase (LArb) and α-glucuronidase (LαGl) further increased xylose yields by 20 percentage points. This study clearly demonstrates that for biomass pretreated by a specific technology (for example, AFEX or similar alkaline pretreatments), it is both possible, and important, to tailor-make specific enzyme cocktails with optimal individual enzyme ratios to achieve higher monosaccharide yields.

This study was carried out at low solids loading (0.2% glucan or 0.58% solids basis, respectively) due to mass-transfer limitations typical for microplate based assays [17]. Therefore, the monosaccharide yield and enzyme loadings discussed here are valid for the best case scenario and there were no significant end-product inhibition or mass transfer limitations typically encountered during high solid loading (15-30% solids loading)-based saccharification. These results should mimic a simultaneous saccharification and fermentation process where the monosaccharides are directly fermented to ethanol without allowing a significant build of sugars. For an industrial high solid loading hydrolysis, more βG and βX might be necessary in order to overcome the build-up of sugar oligomers due to the inhibition of enzymes at high concentrations of monosaccharides and potential unproductive binding to lignin.

AFEX pretreated biomass, which contains nearly all of the original xylan content from untreated biomass, is very different from acid pretreated biomass in terms of its physicochemical composition and enzymatic digestibility [13,15,31]. The efficacy of any pretreatment is normally evaluated by enzymatic hydrolysis at specific conditions (for example, enzyme loading, hydrolysis time, solid loading). In order to achieve an unbiased comparison between pretreated samples, enzymes used for evaluation should be able to reflect the true efficacy of the pretreatment. When comparing AFEX versus dilute acid pretreated biomass, one should include necessary hemicellulase and accessory enzyme activities in the enzymatic cocktail. Otherwise the lack of specific activities could result in underestimating the true digestibility of AFEX pretreated biomass. In addition, for other low severity pretreatments (such as alkaline peroxide) which do not remove most of the hemicellulose fraction, the results in this paper could also be useful for the development of more balanced enzyme cocktails in order to evaluate the pretreatment effectiveness.

## Abbreviations

AFEX: ammonia fiber expansion; βG: β-glucosidase; CBH: cellobiohydrolas; EG: endoglucanase; EX: endoxylanase; LArb: α-arabinofuranosidase; LX: xylanase; PAGE: polyacrylamide gel electrophoresis; PCR: polymerase chain reaction; SDS: sodium dodecyl sulphate.

## Competing interests

The authors declare that they have no competing interests.

## Authors' contributions

DG designed the study, performed the experiments, analysed the results and wrote the manuscript. NU and XY participated in conducting the experiments. SH, KG, PB and DM carried out the molecular cloning and heterologous expression of the bacterial enzymes. VB, SPSC and BED revised the manuscript and coordinated the study. All authors read and approved the final manuscript.

## References

[B1] Jarvis M Chemistry - cellulose stacks up Nature 2003 426 611 612 10.1038/426611a 14668842

[B2] Zhang Y-HP Lynd LR Toward an aggregated understanding of enzymatic hydrolysis of cellulose: noncomplexed cellulase systems Biotechnol Bioeng 2004 88 797 824 10.1002/bit.20282 15538721

[B3] Falkowski P Scholes RJ Boyle E Canadell J Canfield D Elser J Gruber N Hibbard K Hogberg P Linder S The global carbon cycle: a test of our knowledge of earth as a system Science 2000 290 291 296 10.1126/science.290.5490.291 11030643

[B4] Lynd LR Cushman JH Nichols RJ Wyman CE Fuel Ethanol from cellulosic biomass Science 1991 251 1318 1323 10.1126/science.251.4999.1318 17816186

[B5] Jørgensen H Kristensen JB Felby C Enzymatic conversion of lignocellulose into fermentable sugars: challenges and opportunities Biofuels Bioproducts Biorefining 2007 1 119 134

[B6] Ragauskas AJ Williams CK Davison BH Britovsek G Cairney J Eckert CA Frederick WJ Hallett JP Leak DJ Liotta CL The path forward for biofuels and biomaterials Science 2006 311 484 489 10.1126/science.1114736 16439654

[B7] Dale BE Leong CK Pham TK Esquivel VM Rios I Latimer VM Hydrolysis of lignocellulosics at low enzyme levels: application of the AFEX process Bioresource Technol 1996 56 111 116 10.1016/0960-8524(95)00183-2

[B8] Gao D Chundawat SPS Krishnan C Balan V Dale BE Mixture optimization of six core glycosyl hydrolases for maximizing saccharification of ammonia fiber expansion (AFEX) pretreated corn stover Bioresource Technol 2010 101 2770 2781 10.1016/j.biortech.2009.10.056 19948399

[B9] Gao D Chundawat S Liu T Hermanson S Gowda K Brumm P Dale B Balan V Strategy for Identification of novel fungal and bacterial glycosyl hydrolase hybrid mixtures that can efficiently saccharify pretreated lignocellulosic biomass BioEnergy Res 2010 3 67 81 10.1007/s12155-009-9066-6

[B10] Zhou J Wang YH Chu J Luo LZ Zhuang YP Zhang SL Optimization of cellulase mixture for efficient hydrolysis of steam-exploded corn stover by statistically designed experiments Bioresource Technol 2009 100 819 825 10.1016/j.biortech.2008.06.068 18771915

[B11] Mosier NS Wyman CE Dale BE Elander RT Lee YY Holtzapple MT Ladisch MR Features of promising technologies for pretreatment of lignocellulosic biomass Bioresource Technol 2005 96 673 686 10.1016/j.biortech.2004.06.025 15588770

[B12] Lau MW Dale BE Cellulosic ethanol production from AFEX-treated corn stover using *Saccharomyces cerevisiae *424A(LNH-ST) Proc Nat Acad Sci USA 2009 106 1368 1373 10.1073/pnas.0812364106 19164763 PMC2635794

[B13] Chundawat S Beckham G Himmel M Dale B Deconstruction of lignocellulosic biomass to fuels and chemicals Annu Rev Chem Biomol Eng 2011 2 10.1146/annurev-chembioeng-061010-114205 22432613

[B14] Wyman CE Dale BE Elander RT Holtzapple M Ladisch MR Lee YY Mitchinson C Saddler JN Comparative sugar recovery and fermentation data following pretreatment of poplar wood by leading technologies Biotechnol Progress 2009 25 333 339 10.1002/btpr.142 19294662

[B15] Chundawat SPS Vismeh R Sharma LN Humpula JF da Costa Sousa L Chambliss CK Jones AD Balan V Dale BE Multifaceted characterization of cell wall decomposition products formed during ammonia fiber expansion (AFEX) and dilute acid based pretreatments Bioresource Technol 2010 101 8429 8438 10.1016/j.biortech.2010.06.027 20598525

[B16] Wyman CE Dale BE Elander RT Holtzapple M Ladisch MR Lee YY Coordinated development of leading biomass pretreatment technologies Bioresource Technol 2005 96 1959 1966 10.1016/j.biortech.2005.01.010 16112483

[B17] Chundawat SPS Balan V Dale BE High-throughput microplate technique for enzymatic hydrolysis of lignocellulosic biomass Biotechnol Bioeng 2008 99 1281 1294 10.1002/bit.21805 18306256

[B18] Maniatis T Fritsch EF Sambrook J Molecular Cloning, a Laboratory Manual 1982 NY: Cold Spring Harbor Laboratory

[B19] Novagen: pET System Manual 2005 11 Darmstadt: Merck KGaA

[B20] Polizeli MLTM Rizzatti ACS Monti R Terenzi HF Jorge JA Amorim DS Xylanases from fungi: properties and industrial applications Appl Microbiol Biotechnol 2005 67 577 591 10.1007/s00253-005-1904-7 15944805

[B21] Somerville C Bauer S Brininstool G Facette M Hamann T Milne J Osborne E Paredez A Persson S Raab T Toward a systems approach to understanding plant cell walls Science 2004 306 2206 2211 10.1126/science.1102765 15618507

[B22] Shallom D Shoham Y Microbial hemicellulases Curr Opin Microbiol 2003 6 219 228 10.1016/S1369-5274(03)00056-0 12831897

[B23] Saha BC Hemicellulose bioconversion J Indust Microbiol Biotechnol 2003 30 279 291 10.1007/s10295-003-0049-x 12698321

[B24] Kormelink FJM Gruppen H Viëtor RJ Voragen AGJ Mode of action of the xylan-degrading enzymes from *Aspergillus awamori *on alkali-extractable cereal arabinoxylans Carbohydrate Res 1993 249 355 367 10.1016/0008-6215(93)84100-K 8275505

[B25] Banerjee G Car S Scott-Craig JS Borrusch MS Bongers M Walton JD Synthetic multi-component enzyme mixtures for deconstruction of lignocellulosic biomass Bioresource Technol 2010 101 9097 9105 10.1016/j.biortech.2010.07.028 20678930

[B26] Sorensen HR Meyer AS Pedersen S Enzymatic hydrolysis of water-soluble wheat arabinoxylan. 1. Synergy between alpha-L-arabinofuranosidases, endo-1,4-beta-xylanases and beta-xylosidase activities Biotechnol Bioeng 2003 81 726 731 10.1002/bit.10519 12529887

[B27] Benko Z Siika-aho M Viikari L Reczey K Evaluation of the role of xyloglucanase in the enzymatic hydrolysis of lignocellulosic substrates Enzyme Microbial Technol 2008 43 109 114 10.1016/j.enzmictec.2008.03.005

[B28] Bhat MK Hazlewood GP Bedford MR, Partridge GG Enzymology and other characteristics of cellulases and xylanases Enzymes in Farm Animal Nutrition 2001 Oxfordshire: CABI Publishing 11 60

[B29] MP Coughlan GH Hemicellulose and Hemicellulases 1993 Colchester: Portland Press

[B30] Selig MJ Knoshaug EP Adney WS Himmel ME Decker SR Synergistic enhancement of cellobiohydrolase performance on pretreated corn stover by addition of xylanase and esterase activities Bioresource Technol 2008 99 4997 5005 10.1016/j.biortech.2007.09.064 18006303

[B31] Chundawat SPS Donohoe BS Sousa L Elder T Agarwal UP Lu F Ralph JR Himmel ME Balan V Dale BE Multi-scale visualization and characterization of plant cell wall deconstruction during ammonia based thermochemical pretreatment Energy Environmental Sci 2011

